# Colorectal cancer treatment in octogenarians: elective or emergency surgery?

**DOI:** 10.1186/1477-7819-12-386

**Published:** 2014-12-17

**Authors:** Guo Ming-gao, Di Jian-zhong, Wang Yu, Fan You-ben, Huang Xin-Yu

**Affiliations:** Department of Surgery, The Shanghai Sixth People’s Hospital, Shanghai Jiaotong University, Yishan Road 600, 200233 Shanghai, China

**Keywords:** Octogenarians, Colorectal cancer, Surgery, Stents, Outcome

## Abstract

**Background:**

The purpose of this research was to assess the characteristics of octogenarian patients with colorectal cancer and compare specific outcomes due to different types of surgical procedures used to treat the disease.

**Methods:**

A total of 346 octogenarian patients undergoing surgery for colorectal cancer between April 2000 and April 2010 were retrospectively assessed according to elective (n = 261) or emergent (n = 85) admission group. The two groups were compared for clinical variables, surgical procedures, morbidity and mortality, ICU admission, length of hospital stay and overall survival.

**Results:**

The two groups had similar comorbidities. The emergent group had a more advanced Dukes’ stage, higher American Society of Anesthesiologists grading, lower anastomosis rate (40.2 vs 80.1%), higher stoma rate (30.6 vs 9.6%), more complications (71.8 vs 43.3%), nine days longer length of hospital stay and higher (82.4% vs 36.4%) ICU admission rate. Overall mortality was 9.5%, with a higher mortality rate in the emergent group (30.6%) than the elective group (3.1%).

**Conclusions:**

Octogenarians who undergo elective colorectal cancer surgery have better results than those requiring emergent surgery, but both are quite acceptable and we recommend surgical intervention should not be delayed.

## Background

Today, the population is getting older worldwide, with an ever-increasing number of people developing cancer. In this elderly group, colorectal cancer (CRC) is a predominant disease, and about half of all cases occur in patients over 70-years-old. Furthermore, CRC is the second most common cause of cancer death [1].

Treatment of CRC is surgical resection of the primary tumor whenever possible, either for cure or palliation, to avoid late complications, such as obstruction and perforation. Elderly patients may be at risk for later stage and emergent presentation because of insufficient screening, unrecognized symptoms, not deciding whether to receive surgery and inadequate overall access to the healthcare system. Clinical condition is poorer in octogenarian patients with more concomitant illnesses and poorer physiological reserves. Few publications that there are that have reported on outcomes of surgical care of CRC in octogenarian patients have had confused conclusions, and clearly defined guidelines for treatment are lacking [2, 3]. It is difficult for surgeons to decide whether and which surgery is justified in these patients with a limited life expectancy.

Therefore, the purpose of this study was to assess the characteristics in octogenarian patients with CRC. Additionally, we wished to compare specific outcomes due to different types of surgical procedure used to treat CRC in this complex patient group, following elective or emergency surgery.

## Patients and Methods

We studied retrospective data of consecutive elderly patients aged 80 years or over, who were diagnosed with CRC and were admitted to the General Surgery Department of the Shanghai Sixth People’s Hospital between April 2000 and April 2010. A total of 346 patients were included in this study and all received surgical intervention for CRC. The intention of surgery in all cases was curative resection if at all possible. We excluded patients who had endoscopic treatment only. Of these 346 cases, 261 with non-urgent presentations, such as abdominal pain, blood in stool, anaemia, change in bowel habit, mass and weight loss received elective operation and were classed as the elective group. Other 75 patients with acute colonic obstruction and 10 patients with perforation who were classed as the emergent group. Of the patients who presented with acute colonic obstruction, an emergent operation was performed in 64 (85.3%) patients and 11 (14.7%) received self-expanding metal stents (SEMS) as a bridge to surgery. SEMS’ were placed as described previously [4]. All patients’ diagnosis of colorectal adenocarcinoma were histologically proven.

Data regarding the patient’s clinical features, comorbidities, presentation, method of diagnosis, American Society of Anesthesiologists (ASA) grading, location of tumor, type of treatment, Dukes’ staging, treatment outcomes including major 30-day morbidity and mortality, information concerning ICU admission, length of hospital stay and overall survival were collected.

We classified perioperative complications as surgical (bleeding, surgical site infection, anastomotic leak, wound dehiscence and bowel obstruction) or medical (myocardial infarction, deep vein thrombosis, pulmonary embolism, pneumonia, cerebrovascular accident, urinary tract infection, renal failure, multiorgan failure and suffocation). Mortality was defined as that occurring within 30 days postoperatively or before discharge if the patients stayed in the hospital was more than 30 days. Survival was calculated from the time of surgery and survival data were maintained with regular follow-up contact.

All treatment interventions applied in this study including SEMS are officially approved, and are routine therapeutic options for old patients in China. Accordingly, prior to the initiation of the treatments, this work was approved by ethics committee at Shanghai Jiaotong University Affiliated The Six People’s Hospital.

## Results

A total of 346 patients were included in this study. Patient characteristics are shown in Table 1. The median age was 83.5 (range: 80 to 92) years in elective group and 84.7 (range: 80 to 95) years in emergent group. Comorbidities were similar. A significant difference was found in disease presentation, method of diagnosis and ASA grading. Most patients (83.5%) in elective group and 100% patients in emergent group presented with symptoms that prompted a diagnostic evaluation. Of the 261 patients who received elective surgery, 87.4%, 12.6% and 8.8% were diagnosed by colonoscopy, screening colonoscopy and computerized tomography scan, respectively. These conditions were seen in 43.5%, 0.0% and 100.0% of in patients undergoing emergent procedures, respectively. Only 33 (9.5%) patients who did not have any symptoms were diagnosed by screening colonoscopy in both groups. A higher number of high-risk patients (28.2% of ASA stage IV and 3.5% of ASA stage V) were found in emergent group.

**Table 1:** **Characteristics of two groups of octogenarian patients**

Variables	Elective, n (%) N = 261	Emergent, n (%) N = 85	***P***value
Age, median (range) years	83.5(80-94)	84.7(80-95)	
Gender			
Male sex	117(44.8)	36(42.4)	0.69
Comorbidities			
Hypertension	54(20.7)	23(27.1)	0.22
COPD	29(11.1)	13(15.3)	0.03
Chronic heart disease	36(13.8)	17(20.0)	0.17
Cerebrovascular disease	11(4.2)	7(8.2)	0.24
Chronic renal failure	8(3.1)	2(2.4)	1.00
Diabetes mellitus	31(11.9)	10(11.8)	0.98
Clinical presentation			
Asymptomatic	43(16.5)	0(0.0)	<0.01
Symptomatic	218(83.5)	85(100.0)	
Diagnostic examination			
Colonoscopy	228(87.4)	37(43.5)	<0.01
Screening colonoscopy	33(12.6)	0(0.0)	<0.01
Computerized tomography scan	23(8.8)	85(100.0)	<0.01
ASA grading			
I - II	149(57.1)	35(41.2)	<0.01
III	102(39.1)	23(27.1)	
IV	10(3.8)	24(28.2)	
V	0(0.0)	3(3.5)	

Surgical observations are shown in Table 2. A higher number of resection and primary anastomosis (RPA) (80.1% vs 40.2%), and lower number of resection and stoma (RS) (9.6% vs 30.6%) were performed in elective group. The rate of palliative surgery was similar in the two groups. The data show that patients in emergent group presented with advanced Dukes’ stage tumors compared to the emergent group (50.6% C and 24.7% D stage versus 30.3% C and 16.9% D stage).

**Table 2:** **Surgical observations, procedures and tumor characteristics**

Variables	Elective, n (%) N = 261	Emergent, n (%)N = 85	***P***value
Tumor location			
Right	98(37.5)	33(38.8)	0.83
Left and rectum	163(62.5)	52(61.2)	
Treatment			
Curative intent	234(89.7)	61(71.8)	<0.01
Resection + anastomosis	209(80.1)	35(41.2)	<0.01
Resection + ostomy	25(9.6)	26(30.6)	
Palliative intent	27(10.3)	13(15.3)	0.22
Stoma	17(6.5)	10(11.8)	0.60
Bypass	10(3.8)	3(3.5)	
SEMS		11(12.9)	
Dukes’ staging			
Dukes’ A and Dukes B	138(52.9)	21(24.7)	<0.01
Dukes’ C	79(30.3)	43(50.6)	
Dukes’ D	44(16.9)	21(24.7)	

The overall complication rate was significantly higher in the emergent group (71.8% vs 43.3%) (Table 3). Medical complications were more frequent in patients who received emergent surgery (67.0% vs 41.8%) and lower surgical complications were found in the elective group (7.7% vs 15.3%). Emergency was also associated with an increased length of hospital stay and higher rate of admission to ICU. Patients undergoing emergent procedures stayed, on average, nine more days in hospital and 82.4% of patients required ICU admission. The overall mortality was 9.5%, with an obviously higher mortality in emergent group (30.6%) than elective group (3.1%).

**Table 3:** **Comparison of outcomes between elective and emergent surgical procedures**

Outcome	Elective, n (%) N = 261	Emergent, N (%) N = 85	***P***value
Complications			
Overall	113(43.3)	61(71.8)	<0.01
Medical	109(41.8)	57(67.0)	<0.01
Surgical	20(7.7)	13(15.3)	0.04
ICU admission	95(36.4)	70(82.4)	<0.01
Length of stay (days), median (range)	12(4-55)	21(1-92)	<0.01
Mortality	8(3.1)	26(30.6)	<0.01
1-year survival	197(75.5)	37(43.5)	<0.01
3-year survival	121(46.4)	11(12.9)	<0.01

Acute bowel obstruction was the major complication of CRC in octogenarian patients. In emergent group, 75 (88.2%) patients presented with acute colonic obstruction and 11 received SEMS. Table 4 shows the outcomes of SEMS compared to emergent surgery. A lower rate of overall complication (76.6% vs 36.4%) and medical complication (68.8% vs 27.3%) was found. The rate of stoma creation was higher in patients in the emergent group (50.0% vs 9.1%). It appears that mortality was lower in patients who received SEMS treatment (31.3% vs 9.1%), but this data were not significant.

**Figure 1:**
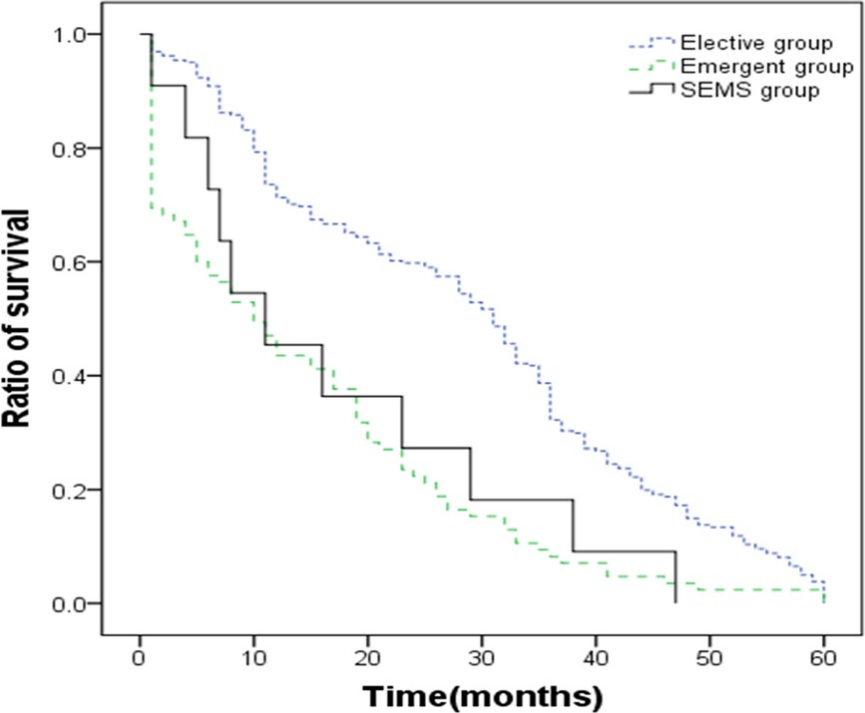
**Kaplan-Meier curves of overall survival for octogenarian patients by procedures.** SEMS, self-expanding metal stent.

Patients were followed up for an average of 36.0 months (range: 2.0 to 60.0 months). The overall median survival was 25.6 months. When overall survival was analyzed by type of procedure, patients in elective group had a median survival of 31.7 months, a one-year survival rate of 75.5% and a three-year survival rate of 46.4%. In comparison, patients in the emergent group had a dramatic decrease in overall median survival of 10.8 months, a one-year survival rate of 43.5% and a three-year survival rate of 12.9%. Patients received SEMS had a similar overall median (11.5 months) and survival rate 90.9% as emergent group (Figure 1).

**Table 4:** **Comparison of outcomes between emergent surgical procedures and self-expanding metal stents**

Outcome	Emergent surgery, n (%) N = 64	SEMS, n (%) N = 11	***P***value
Complications			
Overall	49(76.6)	4(36.4)	0.02
Medical	44(68.8)	3(27.3)	0.02
Surgical	8(12.5)	2(18.2)	0.95
Rate of stoma	32(50.0)	1(9.1)	0.03
ICU admission	49(76.6)	5(45.5)	0.10
Length of stay (days), median (range)	21(1-92)	24(8-40)	
Mortality	20(31.3)	1(9.1)	0.25
1-year survival	28(43.8)	5(60.0)	1.00
3-year survival	7(10.9)	1(10.0)	1.00

## Discussion

Colorectal cancer is a predominantly a disease of elderly people and is a major cause of mortality in the elderly population [2]. Historically, it was suggested that octogenarian patients do not fare well after surgery for CRC, with high rates of emergency presentations, higher postoperative morbidity and mortality. Analysis of several recently published reports shows that elective CRC resection in the elderly population is worthwhile and should be carried out for the same indications as for younger patients [5, 6]. The present study is to assess the characteristics of and compare specific outcomes of elective or emergent surgery in this complex octogenarians group.

As the human body gets old, cumulative physiologic stressors lead to overall decline in organ and tissue function. Physiologic reserves are significantly diminished in octogenarians, and cardiovascular, pulmonary and endocrine comorbidities are common. In a study of 2932 patients ≥ 80 years old with CRC, Frank Marusch *et al*. showed 85% of these patients had a cardiovascular risk factor, and pulmonary comorbidity was found in 25.9% patients [7]. Our study revealed that the rate of having co-existing medical disease was 64.8% in the elective group and 84.8% in emergent group. Hypertension, COPD (chronic obstructive pulmonary diseases) and chronic heart disease were the major comorbidities. This led to high rate of ASA stage ≥ 3 categories, with 42.9% in elective group and 59.8% in emergent group. Additional stress caused by the emergency and later tumor stage led to significantly higher of ASA score in octogenarian patients with emergent presentation.

As previous reports, the current study also revealed an age-related shift toward the colon in the distribution of CRC, and up to 40% of cancers were located in the right colon [8, 9]. Acute mechanical bowel obstruction, which was the most common complication and is a clinical indicator of locally advance disease, was more frequent in octogenarian patients. Glancarlo Basili *et al*. emergency surgery for CRC was carried out in 16% of elderly patients, compared with 4% in the younger group [10]. None of the patients in emergent group were firstly found by colonoscopy screening in our study. Colonoscopy should be recommended as method of choice in octogenarian patients if CRC is suspected. Although the literature concerning screening colonoscopy in the octogenarians is limited, several studies have suggested that a screening colonoscopy may improve poor outcomes by discovering CRC at an earlier stage [11, 12].

As previous reports, for emergent surgery in the unprepared colon, RS is still one of the best operative alternatives in octogenarian patients [13–15]. In our research, a higher number of RPA (80.1% vs 41.2%) and a lower number of RS (9.6% vs 30.6%) were performed in the elective group compared to the emergent group. It is important to consider that the choice of procedure was not randomized, rather, RPA was only carried out when the surgeons believed that local and systemic conditions of the octogenarian patients were appropriate. The high rate of stoma might be attributed to hemodynamically unstable states and lower physiological reserves in the emergent group. RPA was used for 244 out of 346 patients in this study, and 11 (3.2%) of these patients presented with anastomotic dehiscence. There were no differences in anastomotic dehiscence between the elective and emergent group. Compared to 3.0% and 6.3% reported in previous series’ [13, 14], the anastomotic dehiscence rate was not higher in these octogenarian patients. RPA should be encouraged when the over-all physical condition of the patients is appropriate.

Surgery for CRC in octogenarian patients is associated with high morbidity and mortality. The risk of postoperative death rises to 11.9 –38.0% after emergency surgery and 7.4– 11.4% in elective cases [10], making the postoperative mortality rates of present study acceptable. Emergent management of CRC complications, such as obstructing and perforation, remains strongly associated with poorer outcomes in octogenarian patients. The reasons for this are to be seen in a reduced patient status and inadequate preoperative preparation. Ozturk and Yilmazlar reported in a multivariate logistic regression analysis that colorectal disease increased the risk of death by nine-fold in elderly patients undergoing surgery [16]. Emergency surgery, a well-known risk factor, increases operative mortality rates between three-fold- and 10-fold. This research confirm the results of previously published studies, in which morbidity rates of 25– 81% and mortality rates of 17– 30% have been reported [17–20]. Emergency status also caused a significant increase in length of stay and ICU admission.

Compared to elective group, patients had a lower survival; in emergent group, 43.5% versus 75.5% at 1 year and, 12.7% vs 46.4% at 3 years. This survival is also considerably lower than general population after colorectal surgery. These results may partly be explained by the fact that octogenarian patients, especially in emergent group, were more likely to present with a later stage of the disease, were less likely to receive curative surgery, and had higher postoperative mortality. Long-term survival figures could also be affected by the administration of adjuvant chemotherapy or radiotherapy and these treatments may be offered differently according to age. However, most octogenarian patients in this studies did not receive adjuvant treatment.

This retrospective study shows placement of SEMS to be an appealing option as a bridge to semi-elective surgery in octogenarian patients. Compared to emergent surgery, patients treated with SEMS had a significantly lower rate of complications. The mortality rate was 31.3% in the emergent group and 9.1% in patients received SEMS treatment. However, there was no significant difference. This factor may have reached significance if the study population was larger. Among patients who received SEMS, this study showed a marked reduction in stoma formation. SEMS provides a useful alternative to emergent surgical intervention in the management of acute colorectal obstruction for octogenarian patients [4].

The term ‘getting old’ may be defined as ‘an inherent, progressive impairment of function with the passage of time, which cannot be averted, and which causes individuals to become more vulnerable to death’ [21]. But However, it is difficult to define an age limit age that this occurs. As indicated in most previous studies regarding this definition, the physiological and chronic health status of the patient are much more important than the chronological age. Although advancing age is an independent risk factor for postoperative death in elderly patients undergoing colonic resection for cancer [22], in order to prevent high mortality, it should be emphasized that in octogenarians patients who require an operation for colorectal cancer, the operation should be performed as soon as conditions are optimized and should not be postponed. Age should not be a contraindication for surgery. A screening colonoscopy may improve poor outcomes in the octogenarian patients by discovering CRC at an earlier stage. Although emergency operations were associated with a poorer outcome, most patients in our study survived and left the hospital. When emergency of CRC occurs, identifying these high-risk patients and treating them promptly rather than delaying or denying surgery is the best option. SEMS may provide a useful alternative to emergent surgical intervention in the management of acute left-sided colorectal obstruction for octogenarian patients.

## Conclusions

CRC is a predominant disease in elderly patients. Age should not be a contraindication for surgery. Octogenarians who undergo elective surgery have better results than those requiring emergent surgery, but both are quite acceptable and we recommend that surgical intervention should not be delayed.

## Abbreviations

ASA: American Society of Anesthesiologists
CRC: colorectal cancer
COPD: chronic obstructive pulmonary diseases
ICU: intensive care unit
RPA: resection and primary anastomosis
RS: resction and stoma
SEMS: self-expanding metal stents.
